# Current Knowledge on the Diagnostic Methods, Epidemiological Characteristics and Antiviral Strategies of Chicken Anemia Virus

**DOI:** 10.3390/vetsci12121154

**Published:** 2025-12-03

**Authors:** Yuqing Duan, Changchun Gao, Wenlan Cao, Xi Yang, Mengting Zuo, Xiongyan Liang, Yuying Yang, Xiaowei Fang, Kewei Fan, Lei Tan

**Affiliations:** 1 College of Animal Science and Technology, Yangtze University, Jingzhou 434025, China; 2 Fujian Provincial Key Laboratory for Prevention and Control of Animal Infectious Diseases and Biotechnology, Longyan University, Longyan 364012, China; 3 Beijing Huadu Yukou Poultry Industry Co., Ltd., Beijing 102206, China; 4 Hunan Provincial Key Laboratory of the TCM Agricultural Biogenomics, Changsha Medical University, Changsha 410208, China

**Keywords:** chicken anemia virus (CAV), genetic characteristics, epidemiology, diagnosis, antiviral strategies

## Abstract

Chicken anemia virus (CAV) is a single-stranded circular DNA virus classified within the genus *Gyrovirus* of the family *Anelloviridae*. The disease caused by CAV is predominantly characterized by aplastic anemia, lymphatic atrophy, and concurrent immunosuppression. The widespread occurrence of CAV has led to significant economic detriment in the global poultry sector. This review offers a thorough overview of advancements in CAV, encompassing its genomic features and transmission, clinical signs and pathogenicity, diagnostic methodologies, prevalence, and current antiviral strategies, which will provide a valuable resource for future research and the effective management of this pathogen.

## Simple Summary

Chicken anemia virus (CAV), a member of the *Gyrovirus* genus, is a significant pathogen in the global poultry industry. Infection is primarily characterized by aplastic anemia, lymphoid atrophy, and immunosuppression, leading to substantial economic losses. This review provides a comprehensive overview of CAV, covering its genomic structure, transmission, clinical manifestations, pathogenesis, diagnostic techniques, global prevalence, and current control strategies. It serves as a valuable resource for guiding future research and improving the management of this virus.

## 1. Introduction

Chicken anemia virus (CAV), the causative agent of chicken anemia disease, is classified within the genus *Gyrovirus* of the family *Anelloviridae* according to the latest classification [1]. Since the initial isolation of CAV in Japan in 1979, its prevalence and negative impacts on the poultry industry have received adequate attention [2]. In recent years, CAV, along with infectious bursal disease virus (IBDV), Marek’s disease virus (MDV), and avian leukosis virus (ALV), has significantly contributed to immunosuppressive diseases in chickens [3]. In addition to causing immunosuppression, CAV infection leads to severe anemia, weight loss, and generalized lymphoid atrophy in affected chickens [4].

CAV can be transmitted from infected chickens to healthy ones through both vertical and horizontal routes [5,6]. The ability of CAV to be transmitted vertically complicates efforts to eradicate it from poultry populations. Most live attenuated vaccines for chicken infectious diseases are produced using embryonated chicken eggs [7,8]. Reports of CAV contamination in these vaccines are common, contributing to the widespread spread of this pathogen within poultry populations [9,10,11,12].

As a serious immunosuppressive pathogen, co-infections involving CAV and other pathogens are frequently observed, leading to more severe clinical symptoms and pathological changes in the infected animals, which cause substantial economic losses in the poultry industry [13,14]. Recently, the negative impact of CAV on poultry has gained significant attention. Researchers have made extensive efforts to combat this disease, including studying its epidemiology and pathogenicity, as well as developing detection methods and antiviral strategies.

This review provides comprehensive coverage of the current literature concerning CAV, with particular emphasis on its genomic biology, pathogenicity, diagnostic methodologies, and epidemiological characteristics. Furthermore, it will address other topics, such as antiviral strategies. By summarizing the recent advancements in research related to CAV, this review seeks to deepen our understanding of this pathogen and support ongoing research initiatives aimed at addressing this disease.

## 2. Genetic Characteristics and Transmission

### 2.1. Structure and Function of the Encoded Proteins

**Figure 1:**
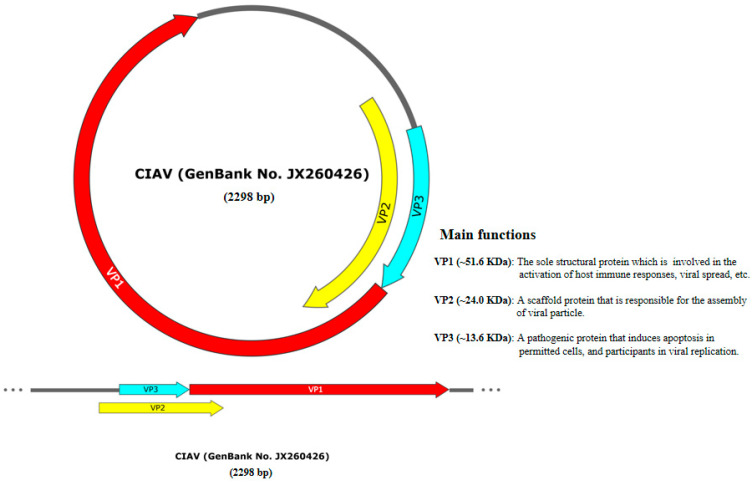
The genomic characterization and protein function of CAV.

CAV is classified as a single-stranded DNA virus that exhibits icosahedral symmetry, with a diameter of approximately 25 nm [1]. The viral genome is nearly 2.3 kbp in length and consists of three overlapping open reading frames (ORFs) that encode the proteins VP1 (~51.6 kDa), VP2 (~24.0 kDa), and VP3 (~13.6 kDa) [15,16] (Figure 1). Among these proteins, VP1 serves as the sole structural protein of CAV and is implicated in a range of biological functions, including the activation of host immune responses [17] and the mediation of viral spread and cell tropism in tissue culture [18]. Recent research demonstrated that CAV VP1 interacted with TBK1, subsequently inhibiting the cGAS-STING signaling pathway [19]. VP2 is regarded as a scaffold protein that is responsible for the assembly of the viral particle. Notably, the antibody titers induced by a formulation of VP1 protein combined with VP2 were significantly higher than those induced by the VP1 subunit protein alone, indicating that the co-formulation of VP1 and VP2 can effectively enhance the immune response in chickens [17,20]. VP3 is a principal viral pathogenic protein that can induce apoptosis in chicken thymic lymphoblasts and primitive hematopoietic cells [21,22]. In addition, the phosphorylation of CAV VP3 has been demonstrated to play a role in viral replication within MSB1 cells [23].

### 2.2. Genotype Characteristics

As a DNA virus, CAV possesses a single serotype. Its genome encodes three ORFs, with the VP1 gene demonstrating a higher rate of genetic variation in comparison to the VP2 and VP3 genes [18,24]. Thus, the VP1 gene has been commonly employed to analyze their phylogenetic relationships [25,26].

Based on the genetic characteristics of the CAV VP1 nucleotide sequences, multiple studies have established that all CAV strains can be classified into four distinct genotypes (I, II, III, and IV) [24,26,27]. Sun et al. reported that a significant majority of CAV strains (89 out of 91) collected in China during the years 2020–2021 were clustered into the sub-genotype IIIa [26]. In a similar vein, Zhang et al. identified 20 CAV strains from Jiangsu Province, along with 42 CAV strains available in the GenBank database, which were categorized into four groups (A–D), with the predominant number falling into group A [24]. Conversely, some researchers have posited that CAV strains can be classified into only three distinct groups or genotypes [5,28]. More recently, Franzo et al. analyzed 1151 VP1 sequences of CAV strains originating from 31 countries between 1974 and 2024 to construct the phylogenetic tree for the purpose of examining genetic evolution [29]. Their findings indicated that all CAV strains were classified into three clades (clade I, II, and III), with the majority of strains belonging to clade II and clade III. Moreover, it was noted that all CAV strains collected globally originated from Asia, with a likely origin in Japan [29].

### 2.3. Frequent Recombinant Events in the CAV Genome

Considering the significant prevalence of CAV infection in avian populations, along with the classification of CAV strains into several distinct genotypes, it is plausible to monitor the co-infections involving CAV strains of different genotypes within a single host or cellular environment. This co-infection may enhance the frequency of recombination events within the CAV genome, thereby accelerating viral genetic evolution, which potentially modifies viral pathogenicity and influences the effectiveness of vaccines [30,31]. A high frequency of recombination events in the CAV genome has been documented in poultry populations [32,33,34,35]. For example, Xu et al. analyzed the genetic characteristics of the complete genomes of 27 CAV strains obtained from 2020 to 2022 in China, revealing that the recombinant events were identified in the genome sequences of five CAV strains [34]. Similarly, Wang et al. reported that recombination had occurred in the complete genomes of two out of twelve CAV strains [35]. It should be highlighted that Li et al. reported the presence of a CAV strain in live-virus vaccines and found that this novel CAV strain might have resulted from the recombination of two distinct CAV strains that belonged to separate genotypes [33]. In addition to findings from China, Dong et al. reported a new recombinant CAV strain designated as Vietnam/PT1/17 in Vietnam in 2019, which is derived from major and minor parental strains belonging to genotype 2 and genotype 3c, respectively [36].

### 2.4. The Transmission of CAV

CAV can be transmitted through both horizontal and vertical routes, with vertical transmission being the main method of spread [12]. The vertical route mainly involves the spread of the virus through egg incubation [7]. Additionally, infectious viruses may be transmitted from CAV-infected roosters to healthy hens during mating, as the CAV genome has been detected in semen samples from roosters [37]. Without maternal antibodies against CAV, chickens are vulnerable to infection via the horizontal transmission route, which primarily occurs through fecal–oral contact when they are exposed to feces, feed, or water contaminated with CAV [4,38]. Furthermore, research has found infectious viruses within the feather shafts of chicks infected with CAV, which can also infect healthy chickens [4]. A recent study showed that *Dermanyssus gallinae* can acquire CAV through biting and transmit the pathogen from infected hosts to healthy chickens via re-biting [39]. These findings suggest that both feathers and *D. gallinae* could play important roles in CAV transmission [4,39].

The prevalence of CAV is influenced by multiple factors, with vertical transmission playing a key role in its spread. Embryonated chicken eggs are commonly employed as vehicles for vaccine production in the poultry industry [7,8]. Due to the widespread occurrence of CAV among chicken populations, including those classified as specific pathogen-free chickens [40]. Instances of CAV contamination have been frequently detected in commercial attenuated vaccines [33,41]. For instance, Li et al. [33] reported that 2 out of 14 batches of live-virus vaccine samples tested positive for the CAV genome. The contaminated CAV exhibited considerable infectivity in chickens, indicating that the presence of CAV contamination in live attenuated vaccines may contribute to the transmission of this infectious agent. To date, CAV contamination has been documented in NDV-attenuated vaccines [9,42], avian encephalomyelitis-attenuated vaccines [43], MDV-attenuated vaccines [43], feline parvovirus-attenuated vaccines [41], attenuated live fowl pox virus vaccines [41], as well as in the combined attenuated vaccines targeting both NDV and infectious bronchitis virus (IBV) [41].

## 3. Clinical Signs and Pathological Changes in CAV Infection

### 3.1. Single Infection

As the natural hosts of CAV, chickens are susceptible to CAV infection regardless of age; however, chicks less than 14 days old are the most vulnerable to this pathogen. The clinical symptoms of CAV infection in chickens are primarily characterized by typical anemia, thymic atrophy, growth retardation, aberrant feathers, bone marrow atrophy, and decreased hematocrit levels [41]. Following CAV infection, pathological changes are observed in the hematopoietic cells of the bone marrow and thymic precursor lymphocytes, marked by lymphatic tissue atrophy and yellowing of the bone marrow. Meanwhile, CAV replicates in the hematopoietic cells of the bone marrow, which destroys the platelet precursor cells, contributing to the damage in the blood coagulation mechanism and resulting in aplastic anemia in chickens [44].

Considering the significant economic losses caused by CAV infection in China, researchers have investigated the outcomes of CAV infection in chickens. Specifically, Wang et al. reported that clinical symptoms such as mental depression and anorexia were monitored at 14 days post-infection challenged with the CAV JS2020-FPV strain; these symptoms were characterized by underwing hemorrhage, fatty marrow, intestinal bleeding, chest muscle bleeding, liver anemia, and death [41]. Moreover, CAV-specific nucleic acids have been detected in the plasma, liver, spleen, and cloaca at 3 days post-infection, while CAV-specific antibodies were detectable at 8 days post-infection in chickens challenged with CAV. Both the nucleic acids and antibodies could still be detected until 40 days post-infection [41]. Additionally, CAV infection significantly reduced the development index of the thymus and bursa in chickens [41]. Another study demonstrated that the challenge of the CAV YN04 strain resulted in significant gross lesions and histopathological changes in the thymus, bursa, and spleen tissues at 14 days post-infection [45]. Additionally, the highest viral loads were detected in the thymus compared to other organs, including the liver, spleen, bursa, and blood [45]. Furthermore, CAV infection led to the indistinct architecture of thymic lobules, as well as necrosis and detachment of the mucosal epithelial cells in the bursal tissues [45].

Yan et al. recently evaluated the pathogenicity of CAV in chicken embryos and found that CAV infection resulted in reduced hatchability rate, decreased embryo weight, and the inhibition of hematopoiesis and T-lymphocyte development [46]. Moreover, the CAV replication was activated before hatching, with the highest virus loads among different tissue samples detected in chicken embryo day 18, thereby contributing to the immunosuppression in hatched chicks [46].

### 3.2. Co-Infection of CAV and Other Pathogens

Given the significant co-prevalence of CAV and other viruses in clinical environments, numerous researchers have investigated the pathogenicity associated with the co-infection of CAV and other viruses in chickens. For example, the administration of an attenuated NDV vaccine with high doses of CAV and FAdV-4 resulted in the development of inclusion body hepatitis-hydropericardium syndrome in chickens, which was characterized by increased mortality rates, reduced growth rates, and more severe tissue lesions in the thymus [9]. Similarly, early co-infection with avian hepatitis E virus (aHEV) and CAV contributed to the increased mortality rates and reduced body weight in specific pathogen-free (SPF) chickens [47]. Moreover, this co-infection significantly exacerbated liver enlargement and thymic atrophy, which in turn facilitated the replication of CAV in the liver, spleen, blood, and thymus [47].

#### 3.2.1. Co-Infection with CAV and GyH1

Yang et al. established a co-infection model of CAV and Gyrovirus homsa 1 (GyH1) in SPF chickens. They demonstrated that co-infection with CAV and GyH1 resulted in severe growth retardation, atrophy of immune organs, lymphocyte exhaustion, and increased mortality in the chickens [48]. Notably, higher viral loads, along with more severe gross lesions and histopathological changes, were observed in various tissue samples from the CAV and GyH1 co-infection group [48].

#### 3.2.2. Co-Infection with CAV and MDV

Two studies have demonstrated that infections caused by MDV and CAV exhibit a synergistic suppressive effect on the immune system, which results in increased mortality rates among SPF chickens [49,50]. Moreover, the CAV-encoded apoptin (VP3) protein has been shown to directly interact with the MDV-encoded Meq protein in chicken embryo fibroblast cells [51]. This interaction may play a significant role in the pathogenic synergies observed during the co-infection with CAV and MDV in chickens [51]. More importantly, Zhang et al. discovered that the CVI988/Rispens vaccination provided effective immune protection against MDV challenge in SPF chickens [52]. However, the introduction of CAV significantly diminished the protective efficacy of CVI988/Rispens vaccine against MDV [52].

#### 3.2.3. Co-Infection with CAV and ALV-J

In vitro experiments have demonstrated that co-infection with CAV and ALV-J enhanced the secretion of inflammatory mediators and induced apoptosis in chicken macrophage-like cells. Moreover, CAV infection has been shown to increase the replication efficiency of ALV in vitro [53]. In vivo experiments indicated that co-infection with CAV and ALV resulted in decreased hatchability of chicken embryos, elevated mortality rates, and inhibited growth in chickens when compared to instances of single infection [54]. Additionally, transcription analysis has revealed that co-infection with CAV and ALV-J significantly suppressed the immune response via regulating the reactive oxygen species and oxidative stress pathways, as well as affecting rRNA metabolism in the spleen samples from infected chickens [55].

#### 3.2.4. Co-Infection with CAV and FAdV E8b

Liu et al. investigated the combined pathogenic effects of the co-infection with CAV and FAdV E8b on SPF chickens and demonstrated that this co-infection significantly increased the clinical scores and mortality rates compared to the single infection with FAdV f8b [56]. Also, co-infection might alter the viral replication efficiency and tissue tropism in chickens [56].

## 4. Current Diagnostic Approaches for the Detection of CAV

The consequences of CAV infection in chickens are primarily characterized by the atrophy of lymphatic organs, the onset of anemia, and immunosuppression; however, it is important to note that overt clinical manifestations are typically absent during the initial stage of the infection. Therefore, early diagnosis of CAV infection is crucial for the development of antiviral strategies against this infectious disease. Recently, a range of diagnostic approaches has been developed for the detection of CAV, which can be categorized into two main types: serological techniques aimed at detecting CAV-specific antibodies and molecular methods designed to assess viral genome sequences. An overview of the current diagnostic approaches for CAV diagnosis, along with their respective characteristics, is presented in Table 1.

### 4.1. Molecular Detection Approaches

In recent years, a variety of molecular diagnostic techniques have been developed for the detection of CAV in clinical samples. These approaches include polymerase chain reaction (PCR) [57], real-time PCR [10,62,78,79,80], dual PCR [58,81], nested PCR [59], droplet digital PCR [60], gene chip assay [64], recombinase polymerase amplification (RPA) [66], recombinase aided amplification (RAA) [67], loop-mediated isothermal amplification (LAMP) [68,69,70], and clustered regularly interspaced short palindromic repeats (CRISPR) and CRISPR-associated (Cas) systems (CRISPR-Cas) [70].

In general, VP1 and VP2 are commonly employed as molecular targets for the development of detection methods aimed at monitoring CAV. Among these diagnostic approaches, conventional PCR assays have been developed for the detection of CAV. The PCR products generated by PCR can also be sequenced for further genetic analysis [6,82]. Real-time PCR technology exhibits higher sensitivity and precision compared to conventional PCR, making it a preferred method for the early detection of CAV infection in clinical settings [10,62,78,80,83]. For instance, Sun et al. established a TaqMan-based real-time PCR method for detecting the presence of CAV, which has a minimum detection limit of 10 copies/reaction, demonstrating a sensitivity that is 100 times greater than that of the conventional PCR method [80]. Given the frequent observation of co-infections involving CAV and other pathogens that have been frequently monitored in clinical settings, multiplex PCR and gene chip assays have been developed to simultaneously detect the presence of CAV and other pathogens. These advancements have significantly enhanced the efficiency of pathogen diagnosis [10,64,83].

In addition to PCR-based diagnostic approaches, various isothermal testing methods have been developed for the detection of CAV, including LAMP [68,70], RAA [67], and RPA [66]. These methods offer several advantages, including enhanced sensitivity and reduced operational time. Furthermore, the implementation of these novel techniques requires only constant-temperature instruments, such as a constant-temperature water bath, which significantly lowers the equipment costs [66]. For example, Wu et al. developed a novel RAA assay targeting the VP2 gene of CAV, with the lowest detection limit reaching 10 copies/reaction. Moreover, this assay could be carried out at 41 °C and completed within 30 min [64].

Recently, Xu et al. successfully developed an RPA method integrated with the CRISPR-Cas13a system [84]. In summary, the CAV VP2 gene sequences were amplified using the RPA technique, and the RPA products were directly detected through the CRISPR-Cas13a system in conjunction with lateral flow assays [84]. This newly established method can be completed within 90 min and has a detection limit of 50 copies/reaction. It is noteworthy that the detection results obtained from this technique demonstrated a 100% (20/20) concordance with the PCR method, suggesting that it may serve as an alternative tool for CAV detection in clinical settings where specialized equipment is not available [84].

### 4.2. Serological Diagnostic Methods

Apart from the molecular diagnostic approaches, a few serological detection methods have been developed for monitoring the presence of CAV-specific antibodies [74,75,85]. Shao et al. synthesized a peptide that recognized the conserved regions of VP1 across various CAV strains [85]. This peptide-based ELISA (pELISA) method exhibited significant specificity for CAV and exhibited high stability. Moreover, the positive detection rate for this novel pELISA method and the commercial ELISA kit (IDEXX) was reported to be 85.7% and 80.4%, respectively [85]. Similarly, Ma and his colleagues successfully expressed and purified three proteins (VP1, VP2, and VP3) of CAV using the baculovirus expression system. The purified CAV recombinant proteins were used to develop an indirect ELISA method, the sensitivity of which was higher than that of the indirect immunofluorescence assay [73]. Furthermore, the detection results of CAV-specific antibodies from this novel ELISA showed a 96.67% consistency rate compared to commercial ELISA kits (IDEXX) [73].

Wanganurakkul et al. purified a 60-amino-acid N-terminally truncated variant of the CAV VP1 protein (designated as Δ60VP1) using an *E. coli* expression system [75]. This recombinant antigen has been employed to establish an indirect ELISA technique for the detection of CAV. The performance of this ELISA demonstrated a high sensitivity of 87.50% and a specificity of 95.31%, with an overall agreement of 90.79% when compared to a commercial ELISA kit [75]. Recently, they have developed an immunochromatographic lateral flow test strip-based assay for detecting the VP1 antigen of CAV [77]. In brief, monoclonal antibodies (MABs) targeting the CAV Δ60VP1 protein were produced and subsequently screened, and two specific MABs (MAB1 and MAB3) were used in the creation of the lateral flow assay [77]. This innovative method demonstrated a detection limit of 625 ng/mL for the Δ60VP1 antigen, which exhibited an accuracy of 92.46% when compared to the real-time PCR method [77].

### 4.3. Other Diagnostic Methods

#### 4.3.1. Virus Isolation

In addition to the aforementioned detection techniques, direct pathogen diagnosis has been recognized as an additional approach to support vaccine development and to advance the understanding of viral infection mechanisms. Among these methods, virus isolation is considered the definitive standard for pathogen detection. Briefly, MDCC-MSB1 cells are commonly used for the isolation of CAV; cells infected with CAV typically display distinct cytopathic effects, characterized initially by cellular swelling, which is subsequently followed by apoptosis [24,27]. However, subsequent experiments should be performed to confirm the presence of CAV-specific antigen or nucleic acids, such as indirect immunofluorescence assay and PCR.

#### 4.3.2. Hematoxylin and Eosin Staining

Histopathology analysis using hematoxylin and eosin (H&E) staining constitutes a crucial technique for identifying histopathological lesions associated with CAV infection. This method facilitates a visual assessment of the cytopathic changes characteristic of lesions induced by CAV. The primary cytopathic changes caused by CAV infection encompassed eosinophilic infiltration and enlargement of the hepatic sinusoidal spaces, as well as congestion and edema within the liver tissues [41]. Moreover, CAV-infected chickens exhibited indistinct architecture or depletion of thymic lobules and the necrotic and exfoliated mucosal epithelial cells in the bursal tissues [45]. Nonetheless, H&E staining is insufficient for the specific detection of histopathological lesions associated with CAV, nor can it confirm the presence of viral antigen or DNA. In light of this, additional experiments, such as PCR and qPCR, are often employed to achieve precise identification of CAV infection [41,45].

## 5. The Global Prevalence of CAV

### 5.1. The Epidemiology of CAV Among the Chicken Industry Worldwide

Since the initial report of CAV infection in Japan in 1979, the occurrences of this pathogen have been subsequently documented in Europe (1981), the United States (1989), South Africa (1991), and Asia (1992) [83,86]. The prevalence of this pathogen has garnered significant attention due to its detrimental effects on the poultry industry. Numerous studies have investigated the prevalence of CAV, mainly in China. In this section, we described the molecular epidemiological characteristics of CAV in China and other countries separately.

#### 5.1.1. The Molecular Epidemiology of CAV Among the Chicken Industry in China

This review encompasses a total of 20,340 tissue or blood samples, the majority of which were obtained from diseased chickens. Among these samples, 3710 were identified as CAV-positive samples, yielding an overall positivity rate of 18.24% (95% CI 17.71–18.77). Specifically, the detection rates of CAV infection varied across different provinces and regions, ranging from 8.04% (95% CI 4.48–11.61) to 58.06% (95% CI 53.53–62.77) (Table 2). The highest rates of CAV detection were recorded in Yunnan Province (58.06%, 95% CI 53.53–62.77), followed by Fujian Province (34.34%, 95% CI 28.84–39.74) and Jiangsu Province (34.24%, 95% CI 30.74–37.73). In contrast, Hubei Province and Henan Province exhibited the lowest detection rates, at 8.04% (95% CI 4.48–11.61) and 8.09% (95% CI 5.83–10.35), respectively.

Based on the clinical symptoms observed in test chickens, the prevalence of CAV was found to be higher in healthy chickens compared to sick or deceased chickens. In addition, several risk factors were identified as being associated with the incidence of CAV within the poultry industry, including the scale of breeding operations, season variations, age, and the specific categories of chickens. For example, the detection rate of CAV infection was significantly higher in chickens from small-scale farms than in those from large-scale farms. Moreover, the infection rates in breeding chickens and commercial laying hens were notably lower than those observed in commercial broiler chickens. Furthermore, the positive rates of CAV infection were significantly elevated in adult chickens compared to chicks.

#### 5.1.2. The Seroprevalence of CAV Among the Chicken Industry in China

A total of seven studies examined the seroprevalence of CAV among chickens in China, which encompassed four provinces or regions and involved 18,536 serum samples collected from healthy chickens. Among these serum samples, 8918 were positive for CAV-specific antibodies, yielding an average positivity rate of 48.11% (95% CI 47.39–48.83). In detail, the seroprevalence of CAV-specific antibodies in chickens from different provinces or regions was 78.13% (95% CI 75.52–80.74) in Shandong Province, 66.8% (95% CI 61.95–71.65) in Zhejiang Province, 39.98% (95% CI 38.96–41.00) in Jiangsu Province, and 33.66% (95% CI 32.08–35.24), respectively. In addition, Xu et al. investigated the seroprevalence of CAV among grandparent chickens from 18 provinces in China and found that the positive farm rate and the average positive individual rate were 97.7% (43/44) and 66.0% (3200/4847), respectively [87]. Importantly, the positive rate of serum samples collected from 14 (77.8%, 14/18) provinces was higher than 60.0% [87].

### 5.2. The Prevalence of CAV in the Chicken Industry in Other Countries

#### 5.2.1. The Seroprevalence of CAV in the Chicken Industry in Other Countries

Apart from China, a total of six studies have investigated the seroprevalence of CAV among chickens in six countries (Supplementary Table S1) [88,89,90,91,92,93], including Bangladesh [88] and India [89,90] in Asia; Canada [91] in North America; and the Central African Republic, Cameroon [92], and Nigeria [93] in Africa. The overall positive rate of CAV antibodies among chickens in the surveyed countries was 67.06% (1340/1985, 95% CI 65.45–69.57). Notably, the average seroprevalence of CAV was 85.22% (392/460) in Bangladesh, 55.68% (206/370) in Nigeria, 62.38% (471/755) in India, 77.06% in Canada, and 36.75% (147/400) in the Central African Republic and Cameroon.

#### 5.2.2. The Molecular Epidemiology of CAV in the Chicken Industry in Other Countries

In addition to these serological studies, various studies have investigated the epidemiological characteristics of CAV in countries such as Vietnam, Greece, Iran, India, and South Korea (Supplementary Table S1) [28,32,36,94,95,96,97,98,99,100,101,102,103,104,105]. For instance, Huynh et al. conducted an investigation into the prevalence of CAV among chicken populations in northern Vietnam in 2018, revealing that 73.4% of the samples (74/119) tested positive for CAV DNA [94]. Moreover, all identified CAV strains were found to be genetically clustered within the genogroups G2 or G3, with no detection of vaccine-like strains [94]. In Poland, Olszewska-Tomczyk et al. demonstrated the classification of the observed field strains into genogroups II and III (IIIa and IIIb) [105].

Apart from chickens, Oh et al. reported that one out of sixteen poultry red mites (Dermanyssus gallinae) tested positive for CAV nucleic acids in Korea [106]. Moreover, a total of 308 ducks were sampled in Laos from 2011 to 2015, of which seven were positive for CAV nucleic acids, resulting in an overall positivity rate of 2.23% [102].

### 5.3. The Co-Infection of CAV and Other Pathogens

In clinical settings, the risk of infection with multiple pathogens is very high in poultry production. Due to the high prevalence of CAV in the poultry industry, coupled with the sub-clinical symptoms exhibited by CAV-infected chickens, the co-infections of CAV and other pathogens have been frequently detected in the Chinese poultry industry. For instance, Li et al. collected 1187 clinical samples from chickens in China, and the presence of CAV and six other viruses was detected using the multiplex RT-qPCR method [107]. The results revealed the highest detection rate of CAV (15.1%), and two co-infection models of CAV+REV and CAV+MDV were identified, with positive rates of 1.6% and 0.4%, respectively [107]. In a similar study, Wang et al. investigated the epidemiological characteristics of CAV and three oncogenic viruses in Jiangsu Province [108]. Results showed that the average detection rates for CAV, MDV, REV, and ALV in 102 clinical samples were 52.94%, 15.67%, 18.63%, and 36.27%, respectively [108]. Notably, the co-infection rates of CAV and three other viruses were 2.0% for CAV+MDV, 4.9% for CAV+REV, and 10.8% for CAV+ALV [108].

### 5.4. The Cross-Transmission Ability of CAV Among Other Species

**Figure 2:**
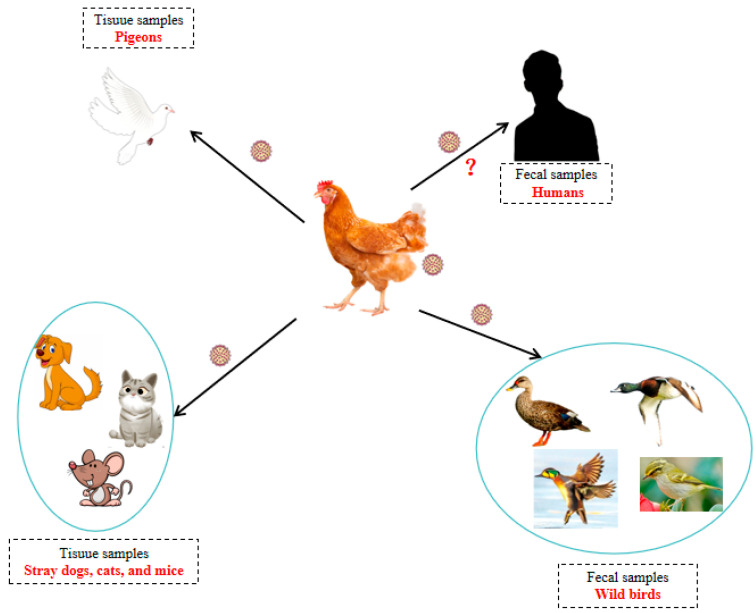
The species potentially susceptible to CAV. (Note: In addition to chickens, infectious viruses or nucleic acids have been isolated or identified in these susceptible species; however, characteristic clinical signs and pathological lesions have not been documented in these species. Furthermore, although nucleic acids of the pathogen have been detected in human fecal samples, it remains to be determined whether this pathogen can naturally infect humans.).

The high prevalence of CAV among chicken populations has significantly heightened the possibility of other susceptible species coming into contact with this pathogen, which potentially contributes to its capacity for cross-transmission. In recent years, the abilities of CAV to infect other species have gathered wide attention in China (as summarized in Figure 2). Yu et al. submitted the first complete genome sequence (GenBank accession no. JQ690762) of a CAV strain isolated from human fecal samples to the GenBank database, while the biological characteristics of this novel strain have not been explored. However, Zhang et al. reported a novel CAV strain (GD-1-12) isolated from a 12-day-old commercial broiler in Guangdong province in 2012; the infection of this novel strain resulted in high mortality, severe anemia, and subcutaneous hemorrhage in chickens [109]. Meanwhile, the genomic sequence of the CAV GD-1-12 strain exhibited higher homology with a CAV strain isolated from human fecal samples [109]. Moreover, Chu et al. conducted a study examining the prevalence of novel viruses in human stool samples in Hong Kong, China [110]. The findings revealed that 13.1% (25 out of 191) of fecal samples from patients experiencing diarrhea tested positive for CAV nucleic acids, suggesting the potential for cross-species transmission of CAV from chickens to humans [110].

Moreover, CAV genomes have been identified in the fecal or tissue samples of other species, including stray mice, stray cats, and stray dogs [111], pigeons [85], wild birds [112], and ducks [102] (Figure 2). Zhang and his colleagues discovered that 10 out of 102 fecal samples collected from stray cats tested positive for CAV nucleotides. Subsequent genetic analysis showed that one variant CAV strain identified in a stray cat may result from the recombination of two distinct CAV strains, namely CAV strains AF311900 and JQ690762 [113]. Research has successfully isolated CAV strains from dogs, mice, and pigeons [72,111]. For example, Shao et al. first isolated one CAV strain from the tissue samples of a sick pigeon (designated as Pigeon-CAV-1906) in China [72]. Further phylogenetic analysis showed that this novel strain was grouped into Group A and exhibited a close genetic relationship with a CAV strain isolated from *Gallus gallus* in Jilin Province, China [72]. Recently, a CAV strain designated as SDTY2021-TJ strain has been successfully isolated from the tissue specimens of a dead vulture in Shandong Province, China [114]. More importantly, this novel strain was highly pathogenic to SPF chickens, which resulted in nearly 50% and 30% mortality rates when challenged with high and low doses of this pathogen [114].

An epidemiological analysis of the prevalence of CAV in wild birds in northeastern China was conducted from 2010 to 2012 [112]. A total of 931 tissue samples were collected from both wild waterbirds and land birds, of which 62 samples tested positive for CAV nucleic acids, resulting in an average detection rate of 6.66% [112]. In addition, the average positive rate of CAV infection among wild waterbirds was 8.87%, while it was 2.49% among land birds. Among various wild duck species, *Anas crecca* exhibited a positive rate of 20.39%, and *Aythya baeri* demonstrated the highest positive rate at 33.33% [112]. Additionally, phylogenetic analysis based on the genetic characteristics of the CAV VP1, VP2, and VP3 genes revealed that 14 CAV strains isolated from wild birds exhibited a closer genetic relationship to the CAV Harbin strain, which had previously been isolated from chickens in China with high pathogenicity [112].

## 6. Antiviral Strategies Against CAV Infection

**Figure 3:**
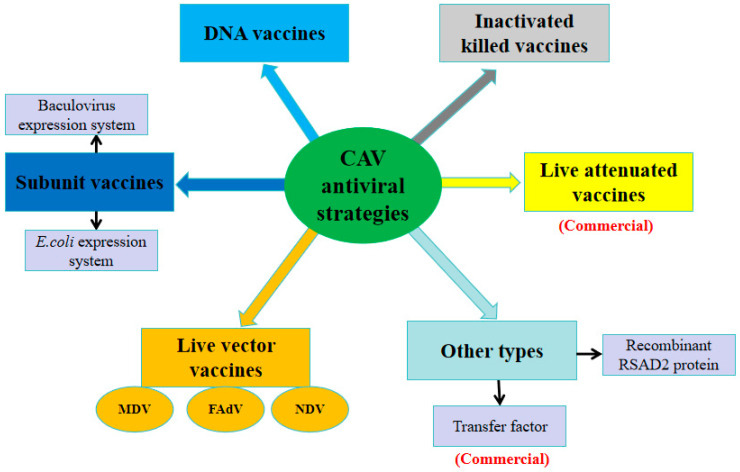
The schematic summary of current anti-CAV strategies.

Vaccine immunization is regarded as the principal strategy for preventing and controlling viral disease [53,115,116]. To effectively control the disease caused by CAV infection, a variety of antiviral strategies have been developed with a primary focus on vaccine development (as summarized in Figure 3 and Table 3).

### 6.1. Vaccines Against CAV Infection

#### 6.1.1. Live Attenuated Vaccines

Live attenuated vaccines have been extensively utilized in the prevention and management of infectious diseases, representing nearly 25% of the global market share for chicken vaccines as of 2022 [115,127,128]. Also, a variety of commercial live attenuated vaccines are available in countries where CAV is prevalent, offering effective protection against field infections caused by CAV [117]. For example, Mckenna et al. demonstrated that administration of the attenuated CAV strains CI34 or CRI18 significantly mitigated severe anemia and reduced lesions in the bone marrow and thymus associated with CAV infection in one-day-old specific-pathogen-free chicks [117]. Although live attenuated vaccines may provide effective protection against CAV infection, careful consideration must be given to the risks of reversion to virulence, their role in viral genome recombination, and the potential for environmental contamination.

#### 6.1.2. Inactivated Killed Vaccines

In addition to live attenuated vaccines, inactivated killed vaccines represent a crucial alternative approach for the prevention of infectious diseases [65], including CIA [118,129]. Zhang et al. conducted an evaluation of the protective efficacy of an inactivated CAV vaccine. Their findings indicated that the administration of this inactivated vaccine resulted in the production of high titers of CAV-specific antibodies in both the vaccinated hens and their offspring [118]. Additionally, these vaccinated hens and their chicks exhibited resistance to the parental CAV strain, showing no clinical manifestations of CIA, including weight loss and thymic atrophy [118].

#### 6.1.3. Subunit Vaccines Developed Using the in Vitro Expression Systems

Viral subunit vaccines are developed utilizing the components of the principal proteins or peptides of viruses using various genetic engineering technologies [121]. Among these genetic approaches, the prokaryotic expression system has been frequently used to produce viral proteins in vitro, due to its high efficiency and low costs. Fang et al. employed the *Escherichia coli* (*E. coli*) expression system to produce the VP1, VP2, and VP3 proteins of CAV. Following this, the immunological effects of these subunit proteins, in conjunction with different immune adjuvants, were evaluated in animal models [17]. The results showed that vaccination with VP1 and VP2 elicited higher antibody titers compared to other groups [17]. Moreover, the combined administration of VP1 and VP2 plus CPG-ODN adjuvant resulted in higher CAV-specific antibody titers than those achieved with the Freund’s adjuvant [17]. Similarly, Liu et al. demonstrated that the combination of recombinant proteins of CAV VP1 and VP2 could effectively stimulate the humoral immune response, as well as promote the proliferation of spleen lymphocytes and enhance cytokine levels [119]. Furthermore, this vaccination system has the potential to decrease the hematocrit levels and viral loads, while simultaneously increasing the thymus index and the survival rate following a challenge with CAV [119].

Shen et al. developed a novel subunit vaccine comprising the recombinant VP1 protein (rVP1) of CAV and pigeon interferon-γ (PiIFN-γ), which was expressed using an *E. coli* expression system [120]. Immunization with the rVP1+PiIFN-γ combination in chickens resulted in higher antibody titers compared to immunization with rVP1 alone or the inactivated vaccine. Conversely, immunization with rVP1+PiIFN-γ significantly reduced hematocrit values in comparison to the other groups [120]. Notably, this combination significantly up-regulated the mRNA expression of IFN-γ while not affecting the expression of IL-4 cytokines in splenocytes of chickens when compared to the other groups [120]. Further investigation is required to determine whether this novel vaccine candidate can be utilized for the prevention and control of CAV infection in chickens.

In addition to the *E. coli* expression system, Tseng et al. employed a baculovirus expression system to develop a subunit vaccine containing both the VP1 and VP2 proteins of CAV [121]. The recombinant VP1 was able to self-assemble into virus-like particles with a diameter of 25 nm [121]. Vaccination with the recombinant VP1, in conjunction with recombinant IL-12 protein, stimulated higher CAV-specific antibody titers compared to other groups, including the group treated with the commercial vaccine [121]. Further analysis demonstrated that the recombinant VP1 subunit vaccine could induce the cell-mediated immune response in vaccinated chickens [121].

#### 6.1.4. Live Vector Vaccines

Co-infections of CAV and other pathogens have been frequently observed in clinical samples within the poultry industry. The development of virus-vector vaccines that express foreign proteins targeting CAV represents a cost-effective strategy to concurrently mitigate the losses associated with these infectious diseases.

MDV is a member of the *Herpesviridae* family and possesses a large DNA genome that includes several non-essential regions for viral replication. This characteristic renders it an excellent viral vector for the expression of foreign proteins [122,123]. Li et al. successfully constructed two recombinant MDV strains that co-expressed the VP1 and VP2 proteins of CAV using different strategies [122]. The recombinant strains exhibited growth kinetics comparable to those of the parent strain in chicken embryo fibroblasts [122]. Furthermore, incubation with the recombinant strains has induced high antibody responses in chickens, which protect against CAV-induced anemia and thymic atrophy [122]. With a similar strategy, Ge et al. employed the CRISPR/Cas9 technology to generate a recombinant MDV strain that expressed the VP1 and VP2 proteins of CAV [123]. Vaccination with this novel vaccine candidate stimulated high levels of CAV-specific antibodies and neutralizing antibodies, significantly suppressing CAV replication in the thymus and reducing the incidence of anemia and thymic atrophy in CAV-challenged chickens [123].

Xu et al. used the CRISPR-Cas9 and Cre-LoxP technologies to generate a recombinant FAdV-4 strain that expressed the VP1 protein of CAV [53]. Notably, this recombinant strain could effectively express the VP1 protein of CAV, while its replication efficiency was much lower than that of the wild-type FAdV-4 strain. However, the antiviral ability of this novel strain against CAV infection has not been analyzed in animal models [53].

Chellappa et al. employed reverse genetic technology to create a recombinant NDV strain R2B that consistently expressed both VP1 and VP2 proteins of CAV (designated as rR2B-FPCS-CAV) [124]. Immunization with this recombinant strain elicited humoral immune and cell-mediated immune responses against CAV that were comparable to those observed in the group vaccinated with the inactivated CAV vaccine [124]. Further animal studies demonstrated that this innovative vaccine candidate conferred partial protection against virulent NDV challenges in chickens and significantly reduced the viral loads of CAV in the blood samples of chickens exposed to CAV [124].

#### 6.1.5. DNA Vaccines

A DNA vaccine consists of plasmid DNA, which is derived from bacteria and contains one or more foreign genes, along with eukaryotic promoter and regulatory gene elements [119]. DNA vaccines can stimulate both humoral and cellular immune responses and induce the production of T lymphocytes with cytotoxic functions [119]. Liu et al. successfully constructed a eukaryotic expression plasmid that expresses both the VP1 and VP2 proteins of CAV. Intramuscular injection of this novel DNA vaccine effectively stimulated both humoral and cellular immune responses in two-week-old SPF chickens [119]. Additionally, this vaccine has been shown to provide partial protection against CAV infection. Moreover, the efficacy of this DNA vaccine was significantly enhanced when in conjunction with the subunit vaccine that contained both the VP1 and VP2 proteins [119].

#### 6.1.6. Alternative Antiviral Approaches

Antiviral activity of recombinant chicken RSAD2

Radenosine methionine domain protein 2 (RSAD2), also known as viperin, is a significant interferon-stimulated gene that plays a crucial role in innate antiviral immunity [130]. The antiviral activities of RSAD2 have been confirmed across various viral species in recent years [131,132]. Zhang et al. successfully expressed chicken RSAD2 using a pFastBac expression vector. Treatment with the purified RSAD2 significantly reduced the viral loads of CAV in MDCC-MSB1 cells compared to the control group [125]. Further animal experiments demonstrated that immunization with this novel protein alleviated immune organ atrophy and hematocrit levels caused by CAV infection in chickens; meanwhile, this treatment further decreased viral loads in various organs [125]. Furthermore, the antiviral activity of this protein has been significantly enhanced when used in conjunction with the recombinant VP1 and VP2 proteins expressed within the same system [125].

Antiviral activity of recombinant chicken transfer factor

Transfer factor is a type of small, permeable molecule secreted by immunologically active T lymphocytes in animal leukocytes [126]. Since its initial discovery in the 1950s, various biological characteristics of transfer factor have been confirmed, including its role in regulating immunity and its participation in the development of the intestinal mucosal barrier [126].

Miao et al. investigated the effects of transfer factor on the pathogenicity of CAV in SPF chickens [126]. The results demonstrated that treatment with both low and high doses of transfer factor significantly up-regulated the transcription of IL-12, Mx, and IFN-γ in peripheral blood lymphocytes and increased the proportions of CD4+ and CD8+ T cells in the chickens [126]. Additionally, transfer factor treatment also alleviated the spleen enlargement and thymus atrophy caused by CAV infection and significantly inhibited the viral replication in the blood, liver, and thymus [126]. Moreover, the antiviral activity in the high-dose group was enhanced compared to the low-dose group [126].

## 7. Prevention and Eradication

The widespread occurrence of CAV poses a significant threat to the global poultry industry. The elimination of this infectious disease has garnered considerable attention, primarily focusing on the optimization of farm management practices. These strategies include the intensification of environmental disinfection procedures, encompassing the use of chemical disinfectants (1% glutaraldehyde and 0.4% beta-propiolactone) [133], the improvement of the feeding and overall management standards, and the enforcement of stringent flock quarantine measures. It has been reported that anti-CAV antibodies could be transferred from hens to their chicks through the egg yolk, thereby providing protection to the chicks against field CAV infection [40]. Fujiwara et al. successfully eliminated the early invasion of CAV in SPF chicken flocks in Japan [134]. Briefly, they gathered eggs from CAV-infected hens and used semen that was devoid of CAV for artificial insemination. Subsequently, the fertilized eggs were incubated in a separate facility that was also free from CAV, leading to the production of SPF chicks [134]. Both horizontal and vertical transmission pathways are capable of disseminating CAVs. The application of chemical disinfectants, along with the surveillance of CAV nucleic acids or specific antibodies, is also essential for the effective management and eradication of this infectious disease [134].

## 8. Perspective and Future Directions

Since the initial documented instance of CAV infection reported in Japan in 1979, this infectious agent has exhibited a widespread presence worldwide. Nevertheless, the implementation of prevention and control strategies to combat this disease is often overlooked, as evidenced by the limited number of related publications available in both Chinese and English databases. At present, CAV has been acknowledged as one of the most significant immunosuppressive diseases, resulting in considerable economic losses within this domain. Hence, lots of efforts have been made for the prevention and control of this infectious disease, which mainly include investigating its epidemiological characteristics and creating diagnostic approaches, as well as developing antiviral technologies.

At present, the majority of studies on CAV are conducted in China, focusing on the investigation of its epidemiological characteristics, the advancement of detection approaches, and the formulation of antiviral strategies. There are several reasons contributing to this trend. First, China has cultivated a substantial population of chickens, second only to the United States in terms of poultry production. Second, various farming models for poultry rearing are present in China. Notably, free-range and small-scale farming systems characterized by poor management practices contribute to the increased incidence of CAV within poultry populations. Thirdly, in addition to chicken, the incidence or prevalence of CAV among its susceptible species has garnered considerable attention in China. However, whether this pathogen can naturally infect these species remains to be elucidated through further research. Fourth, as the standards of feeding management in the poultry industry in China continue to progress, measures aimed at eradicating various infectious agents, including ALV, NDV, and MDV, are being implemented. In recent years, there has been a growing focus on the prevention, management, and even eradication of CAV within Chinese poultry breeding enterprises.

There are multiple research gaps considering the advancement in CAV. Firstly, the existing global research concerning the prevalence of CAV is limited. Further investigations should focus on the epidemiological characteristics of CAV in these nations, with particular emphasis on recombination events occurring between vaccine and field strains, especially in areas engaged in the importation or exportation of poultry. Secondly, there is no established consensus regarding the viral genotype at present, highlighting the need for the identification and selection of suitable molecular markers and bioinformatics software for future genotyping efforts. Thirdly, instances of CAV infection in various species have been reported in China. However, it remains unclear whether CAV can naturally infect these mammalian species, as well as the potential molecular mechanisms involved in its cross-species transmission. Fourthly, numerous diagnostic techniques have been developed, the majority of which are still in the experimental phase and require evaluation of their feasibility and accuracy in practical applications. The breeding site is usually located in remote areas. The development of on-site detection methods, such as lateral flow assay [77] and CRISPR-based detection methods [84], will meet the preliminary screening of on-site samples. In addition, a variety of commercial live attenuated vaccines have been developed for the prevention and control of CAV infection; novel molecular or serological detection methods need to be created for distinguishing vaccination from natural infection. Fifthly, various types of vaccines targeting CAV infection have been created [135]. Only live attenuated vaccines are accessible in certain countries, while they are absent in China. Thus, there is an urgent need for the development of alternative effective vaccines or agents, which will facilitate the management and potential eradication of this disease in the future. Finally, it is imperative to identify the cellular factors involved in CAV infection. Developing gene-edited chickens that are resistant to CAV infection could represent a novel approach for the management of this infectious agent.

## 9. Conclusions

In conclusion, CAV has been highly prevalent in the poultry industry, which poses a significant threat to the global poultry industry. Additionally, the cross-transmission capability of this pathogen from chickens to other species has been taken into consideration. However, effective strategies to combat CAV infection are still limited. In light of this, there is a need to develop early, rapid, and accurate diagnostic methods that are cost-effective for detecting CAV, which will be beneficial for monitoring the epidemiological characteristics of this pathogen, particularly among species susceptible to CAV. Furthermore, the development of alternative antiviral strategies against CAV infection should be considered in the future. These strategies may include the creation of gene-edited chickens that are resistant to CAV infection, aimed at minimizing the economic losses caused by this disease.

## Figures and Tables

**Table 1:** Representative diagnostic methods for the detection of CAV.

Target	Approaches	Targeted Gene (CAV)	Main Characteristics	References
CAV nucleotide	PCR	VP1	The sensitivity was 11 copies/μL.	[57]
	Dual PCR	VP1	Simultaneously monitored the presence of CAV and FAdV-4, with detection limits of 48 fg/μL and 64 fg/μL, respectively.	[58]
	Nest PCR	Not mentioned	The detection limit of this novel approach was 100 pg/μL and 1 fg/μL for the first and second amplification, respectively.	[59]
	DdPCR	VP2	The lowest detection limit was 2.4 copies/μL of CAV plasmid or CAV contamination at 0.1 EID50/1000 feathers in attenuated vaccines.	[60]
	Real-time PCR	VP2	The detection limit was 5.33 copies/μL, which was 1000 times greater than the traditional PCR (5330 copies/μL).	[61]
		VP2	The sensitivity was 6.36 copies/μL, which was 1000 times greater than the traditional PCR (6360 copies/μL). Furthermore, the detection limit was 1 EID50 in commercial poultry vaccines.	[62]
		VP1	The sensitivity was 21 copies per reaction; the R2 value and efficiency of amplification were 0.987~0.994 and 93%~102%, respectively.	[10]
		VP1	The lowest detection limit was 0.01 fg/μL or 820 copies/μL of the plasmid DNA.	[10]
	MRT-qPCR	Not mentioned	This novel method can simultaneously detect the presence of CAV, MDV, REV, ARV, IBDV, and FAdV in clinical setting.	[63]
	Gene chip	VP2	The sensitivity of gene chip for CAV diagnosis was 1000 copies/μL, which can simultaneously monitor the presence of CAV, REV, ALV-A, ALV-C, and ALV-D.	[64]
	RPA combined with CRISPR/Cas13a and a lateral flow strip	VP2	The detection limit of this novel approach was 50 copies/μL; the results could be obtained within 1.5 h and monitored by naked eye.	[65]
	Real-time fluorescence RPA	VP2	The sensitivity of this method was 100 copies/μL; the detection result could be obtained within 30 min under 39 °C constant temperature.	[66]
	RAA	VP2	The lowest detection limit was 10 copies/μL; the assay could be carried out at 41 °C and completed within 30 min.	[67]
	LAMP	VP1	The detection limit was 749 copies/μL, 648 copies/μL, and 307 copies/μL for CAV, FAdV-4, and ChPV, respectively.	[68]
		VP2	This method exhibited similar sensitivity to qPCR method, with a detection limit of 65 copies/reaction.	[69]
		VP1	The detection limit was 50 copies/μL, 16 copies/μL, 20 copies/μL, and 250 copies/μL for CAV, REV, MDV, and IBDV, respectively.	[70]
			The detection limit was 100 fg per reaction, which was 100 times higher than the conventional PCR.	[71]
CAV antibody	pELISA	VP1	This method specifically reacted with serum against CAV, while the positive and negative coincidence rates of this method compared to the commercial ELISA kit (IDEXX) were not high.	[72]
	Indirect ELISA	VP1/VP2/VP3	The sensitivity of this method was higher than that of the IFA method; this method demonstrated a 96.67% positive coincidence rate when compared to the commercial ELISA kit (IDEXX).	[73]
		VP2/VP3	The VP2 and VP3-based ELISAs exhibited sensitivities of 97.5% and 85%, respectively.	[74]
		VP1	The agreement between this indirect ELISA method and the commercial ELISA kit was 90.79%.	[75]
		CAV crude virus	The relative sensitivity, specificity, and accuracy were 93%, 78%, and 86%, respectively. The agreement between commercial and this method was 0.71.	[76]
CAV antigen	Lateral flow assay	VP1	This innovative method demonstrated a detection limit of 625 ng/mL for the Δ60VP1 antigen	[77]
	Virus isolation	virus	This method is considered the definitive standard for pathogen detection, but it is technically demanding and time-consuming.	[24,27]
	H&E staining	Not targeting viral gene	This method could only detect the pathological changes caused by CAV infection, thus exhibiting low specificity and sensitivity.	[41]

PCR: Polymerase chain reaction; DdPCR: Droplet-based digital PCR; RAA: Recombinase aided amplification; RPA: Recombinase polymerase amplification; LAMP: Loop-mediated isothermal amplification; H&E staining: Hematoxylin and eosin staining.

**Table 2:** Pooled molecular prevalence of CAV infection in China.

		No. of Studies	No. of Tested Samples	No. of Positive Samples	Heterogeneity			% (95 CI)
					χ^2^	*p*-Value	I^2^ (%)	
Regions	Hainan	1	3560	930	-	-	-	26.12% (24.68–27.56)
	Jiangxi	1	50	9	-	-	-	18.0% (7.35–28.64)
	Shandong	6	3936	631	49.714	<0.001	89.94%	16.03% (14.88–17.18)
	Henan	1	557	45	-	-	-	8.09% (5.83–10.35)
	Jiangsu	3	701	240	28.602	<0.001	93.00%	34.24% (30.74–37.73)
	Guangxi	1	350	60	-	-	-	17.14% (13.19–21.09)
	Fujian	1	297	102	-	-	-	34.34% (28.94–39.74)
	Hubei	1	224	18	-	-	-	8.04% (4.48–11.61)
	Yunnan	1	422	245	-	-	-	58.06% (53.53–62.77)
Clinical symptoms	Sick chickens	21	14,589	2563	1.322	0.250	0	17.57% (16.95–18.19)
	Healthy chickens	3	4852	1087	21.548	<0.001	90.72%	22.40% (21.23–23.57)

**Table 3:** Summary of antiviral strategies against CAV infection.

	Antigens	Expression System	Modification	Route	Results	References
Live attenuated vaccines	Whole virus	/	/	Intramuscular injection	Significantly mitigated severe anemia and reduced lesions in the bone marrow and thymus associated with CAV infection in chickens.	[117]
Inactivated vaccines	Whole virus	/	/	Intramuscular injection	These vaccinated hens and their chicks exhibited resistance to the parental CAV strain.	[118]
Subunit vaccines	VP1 and VP2	*Escherichia coli*	/	Intramuscular injection	Induced higher antibody titers compared to other groups.	[17]
	VP1 and VP2	*Escherichia coli*	/	Intramuscular injection	Stimulated the humoral immune response, decreased the hematocrit levels and viral loads in CAV-infected chickens.	[119]
	VP1	*Escherichia coli*	Combined with interferon-γ	Intramuscular injection	Induced higher antibody titers compared to other groups, including the inactivated vaccine group.	[120]
	VP1 and VP2	Baculovirus/Sf9 cells	Combined with IL-12 protein	Intramuscular injection	Induced the cell-mediated immune response and high CAV-specific antibody.	[121]
Live vector vaccines	VP1 and VP2	MDV	/	Subcutaneous injection	Inhibited CAV replication in thymus and alleviated thymic atrophy caused by CAV infection in chickens.	[122,123]
	VP1	FAdV-4	/	/	This recombinant strain effectively expressed CAV VP1.	[53]
	VP1 and VP2	NDV	/	Intramuscular injection	Elicited high humoral immune and cell-mediated immune responses, and provided partial protection against CAV infection in chickens.	[124]
DNA vaccines	VP1 and VP2	Eukaryotic expression plasmid	/	Intramuscular injection	Induced humoral and cellular immune responses and provided partial protection against CAV infection in chickens.	[119]
Other types	RSAD2	Baculovirus/Sf9 cells	/	Intramuscular injection	Alleviated immune organ atrophy and hematocrit levels, decreased viral loads in various organs in chickens.	[125]
	Transfer factor	Commercial	/	Orally	Enhanced the immunity system in chickens, alleviated spleen enlargement and thymus atrophy, and reduced viral loads in various organs of CAV-challenged chickens.	[126]

## Data Availability

No new data were created or analyzed in this study. Data sharing is not applicable to this article.

## Supplementary Materials

The following supporting information can be downloaded at https://www.mdpi.com/article/10.3390/vetsci12121154/s1, Table S1: Pooled molecular and serological prevalence of CAV infection in foreign countries.
