# Supplementary material for: Current Knowledge on the Diagnostic Methods, Epidemiological Characteristics and Antiviral Strategies of Chicken Anemia Virus
Source: Vet Sci. 2025 Dec 3;12(12):1154. doi: 10.3390/vetsci12121154 (PMC12737574; doi:10.3390/vetsci12121154)
Supplement: Supplementary file 1 [file vetsci-12-01154-s001.zip › vetsci-3946674-supplementary.pdf]

**Supplementary Table S1:** Pooled molecular and serological prevalence of CAV infection in foreign countries.

| | County (Investigated year) | Species | No. of samples | No. of positive samples | Positive rate/% | Genetic characteristics | References |
|---|---|---|---|---|---|---|---|
| Serological survey | Bangladesh (2020) | Chicken | 460 | 392 | 85.22 | Not determined | [88] |
| | India (before 2021) | Chicken | 404 | 351 | 86.88 | Not determined | [89] |
| | India (2015-2018) | Chicken | 120 | 66 | 55.0 | Not determined | [90] |
| | Canada (2010) | Chicken | 231 | 178 | 77.06 | Not determined | [91] |
| | Central African Republic and Cameroon (2008-2010) | Chicken | 400 | 147 | 36.75 | Not determined | [92] |
| | Nigeria (before 2004) | Chicken | 370 | 206 | 55.68 | Not determined | [93] |
| Molecular survey | Vietnam (2018) | Chicken | 119 | 74 | 62.2 | G2 and G3 | [94] |
| | Vietnam (2020-2021) | Chicken | 47 | 20 | 42.55 | G2 and G3 | [95] |
| | Vietnam (2016-2018) | Chicken | 330 | 157 | 47.58 | G2 and G3 | [96] |
| | Iran (2017) | Chicken | 40 | 13 | 32.5 | Highly homologous to Egyptian strains | [97] |
| | Iran (before 2013) | Chicken | 100 | 46 | 46.0 | A novel recombinant strain was identified, derived from a Chinese strain and a Iran strain | [98] |
| | India (2016-2017) | Chicken | 65 | 39 | 65.0 | Not determined | [90] |
| | India (2018-2019) | Chicken | 443 | 81 | 20.1 | Not determined | [99] |
| | South Korea (2009) | Chicken | 178 | 170 | 95.51 | G2 and G3, and some strains showed high similarity to the vaccine strain (26P4) | [28] |
| | Japan (2023) | Chicken | 9 | 9 | 100.0 | G3 | [36] |
| | Japan (2023) | Chicken | 3 | 1 | 33.33 | Not determined | [100] |
| | Japan (2017) | Chicken | 3 | 3 | 100.0 | G3 | [101] |
| | Laos (2011-2015) | Chicken | 297 | 54 | 18.1 | Not determined | [102] |
| | Argentina (2007) | Chicken | 31 | 18 | 58.06 | Not determined | [103] |
| | South Africa (2006) | Chicken | 198 | 49 | 24.75 | Genetically grouped with the CAV genotype D2 isolated from Japan, USA, and Malaysia. | [104] |
| | Central African Republic and Cameroon (2008-2010) | Chicken | 144 | 39 | 27.08 | G2 and G3 | [92] |
| | Egypt (No information) | Chicken | 42 | 19 | 45.23 | These CAV strains were divided into 4 groups. Several potential recombination events were detected in some novel strains, which occurred between the vaccine and field strains. | [32] |
| | Poland (2013) | Chicken | 106 | 16 | 15.09 | These strains belonged into the groups II and III | [105] |
